# Effect of Surface Charge on the Fabrication of Hierarchical Mn-Based Prussian Blue Analogue for Capacitive Desalination

**DOI:** 10.1021/acsami.2c08192

**Published:** 2022-08-25

**Authors:** Xingyan Zhang, Esteban Alejandro Toledo-Carrillo, Dongkun Yu, Joydeep Dutta

**Affiliations:** Functional Materials, Department of Applied Physics, School of Engineering Sciences, KTH Royal Institute of Technology, Hannes Alfvéns Väg 12, 11419 Stockholm, Sweden

**Keywords:** Mn-based Prussian blue analogue, carbon cloth electrode, surface charge, electronegativity, capacitive deionization, desalination

## Abstract

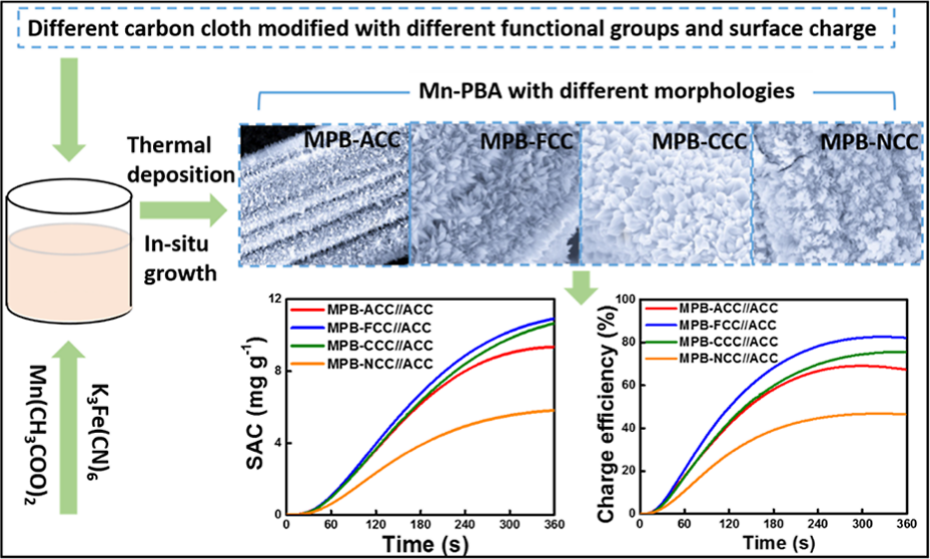

Multiple and hierarchical manganese (Mn)-based Prussian blue analogues obtained on different substrates are successfully prepared using a universal, facile, and simple strategy. Different functional groups and surface charge distributions on carbon cloth have significant effects on the morphologies and nanostructures of Mn-based Prussian blue analogues, thereby indirectly affecting their physicochemical properties. Combined with the advantages of the modified carbon cloth and the nanostructured Mn-based Prussian blue analogues, the composite with negative surface charge formed by the electronegativity differences shows good electrochemical properties, leading to improvement in charge efficiency during capacitive desalination. An asymmetric device fabricated with Mn-based Prussian blue analogue-modified F-doped carbon cloth as the cathode and acid-treated carbon cloth as the anode presents the highest salt adsorption capacity of 10.92 mg g^–1^ with a charge efficiency of 82.28% and the lowest energy consumption of 0.45 kW h m^–3^ at 1 V due to the main influencing factor from the negative surface charge leading to co-ion expulsion boosting the capacitive deionization performance. We provide insights for further exploration of the relationship between second-phase materials and carbon cloth, while offering some guidance for the design and preparation of electrodes for desalination and beyond.

## 1 Introduction

With the increasing global population and the rapid industrialization of the world, a reliable clean freshwater supply is crucial, which has become an urgent problem for sustainable development.^1,2^ To solve this problem, various desalination technologies, such as thermal distillation, reverse osmosis, electrodialysis, etc., have been developed to produce freshwater from brackish water or seawater.^3,4^ However, the high energy consumption in these processes limits their wider application. Capacitive deionization (CDI) is considered one of the new technologies with promise that make use of enhanced adsorption of ions upon the application of a low voltage to transport ions from brackish water to the electrode/solution interface for desalination, leading to much lower energy consumption as well as lower secondary pollution.^5,6^ Considerable progress in the last decade has been made in the development of electrode materials, the configuration of devices, process analysis, etc., but its low desalination capacity still limits its practical application and further development.^7,8^ Thus, to expand the scale of application of CDI for water desalination, it is important to enhance the salt adsorption capacity of the electrode materials.

Carbon materials, owing to their native advantages such as good electrical conductivity, high specific surface area, lower cost, amongst others, are often the most preferred electrodes in capacitive deionization devices.^9,10^ Carbon cloth shows excellent performance due to its acceptable electrical conductivity and flexibility for adjusting the electrodes in complex devices of any designed shapes.^11,12^ However, it mainly relies on the physical adsorption of ions on the electrode surface that forms an electric double layer during desalination, which results in a slowing down of the adsorption processes, thus limiting total salt adsorption capacity. Thus, researchers have explored different strategies to improve the electrochemical behavior of carbon cloth electrodes.^13−15^ Surface modification of carbon electrodes is an obvious method to improve the physicochemical properties, like electrical conductivity, surface activity, and specific surface area.^16−18^ A more promising approach is by combining carbon materials with other materials with pseudocapacitive properties that improve surface ion adsorption. Furthermore, the introduction of the reversible redox reaction of the second material can produce additional desalination capacity due to the intercalation of salt ions, thereby enhancing the overall electrochemical performances and improving the salt adsorption efficiency of the hybrid electrode. Recently, many composite carbon cloth electrodes with materials like metal–organic framework (MOF),^19^ manganese dioxide (MnO_2_),^20^ cobalt ferrite/ferric oxide (CoFe_2_O_4_/Fe_2_O_3_),^21^ and reduced graphene oxide-polypyrrole-manganese dioxide (rGO-PPy-MnO_2_)^22^ have been reported to be advantageous as capacitive electrodes for desalination. These hybrid electrodes show better specific capacitance and higher charge efficiency, which demonstrates that the introduction of nanomaterials is an effective strategy to significantly improve the adsorption capacity of porous carbon electrodes.

Prussian blue analogues (PBAs), as one such metal–organic framework material, have an open-framework structure providing fast transmission channels for ions, thereby enhancing the ion adsorption.^23−25^ However, the use of PBAs in capacitive desalination devices is still severely limited due to their poor electrical conductivity, although it is widely used in catalysis, energy storage, magnetism, and luminescence applications.^26−29^ Therefore, combining PBA, like the Mn-based Prussian blue analogue (MPB) as a potential candidate, with highly conductive materials, is one of the effective approaches to boost the electrochemical performances. Huang et al. obtained manganese oxide/manganese ferrocyanate (MnO_*x*_-MHCF) composites by in situ self-transformation during synthesis and reported boosted electrochemical performances of supercapacitors.^30^ Zhao et al. prepared potassium manganese hexacyanoferrate/graphene nanocomposites by ball-milling, and these materials showed higher initial discharge capacity, average working potential, rate capability, and longer cycle life for potassium-ion battery due to the smaller sizes of the Prussian blue analogue crystals and the high conductivity rendered by the graphene admixture.^31^ Cao et al. reported that potassium ions stabilized hollow Mn-based Prussian blue analogues with stable structure and enhanced sodium ion transport kinetics in the cathodes of sodium-ion batteries.^32^ In addition, surface modification or defect control is also an approach reported to improve the electrochemical performances of PBAs. Yang’s group introduced cation (Mn) vacancies in the surface of Mn–Fe Prussian blue analogues, leading to reversible phase transitions to deliver long-term cycling stability for sodium-ion storage.^33^ Furthermore, it is well known that the distribution and size of the second-phase materials on the carbon cloth can effectively enhance the electrochemical behaviors of the electrode.^34^ A relatively smaller particle size can increase the surface area of the hybrid electrode to improve its adsorption capacity; well-dispersed material in the carbon matrix leads to more uniform electrochemical potential distribution on the electrodes. However, due to the limited surface activated sites and weak interaction between metal ions and carbon cloth, aggregation of nanoparticles usually occurs on the surface of the carbon cloth, leading to a non-uniform electric field distribution.^35^ Therefore, a feasible strategy is to increase the surface-active sites by modifying the surface of the carbon electrodes for enhancing the interaction with metal ions and the substrate. Meanwhile, it is still a challenge to develop a simple and effective method to synthesize composites of different substrates and Prussian blue analogues with good interfacial adhesion.

In this work, hierarchical Mn-based Prussian blue analogue (MPB) was successfully synthesized and applied on various substrates through a universal, simple, and facile thermal adsorption/deposition approach. The synergistic effect between the nanostructured MPB and the modified carbon cloth, especially the negative surface charge distribution caused by the electronegativity, improves the electrochemical performance of the hybrid electrodes. Asymmetric devices fabricated in a flow-through architecture presented high salt adsorption capacity and charge efficiency at a relatively low voltage (1.0 V) showing promising application potential in water desalination.

## 2 Experimental Section

All the analytical grade reagents (Sigma-Aldrich), acetone (BASF, Germany), ethanol (Solveco, Sweden), concentrated hydrochloric and hydrofluoric acid (Sigma-Aldrich), and concentrated nitric (Merck, Germany) used in the experiment were purchased and directly used without further purification.

### 2.1 Pretreatment of Various Substrates

The as-purchased carbon cloth (CC, Zorflex FM10, Chemviron Carbon Ltd., UK) was cleaned by sequential sonication in acetone, ethanol, and deionized (DI) water for 30 min, respectively, followed by drying at 60 °C overnight in an atmospheric oven (Memmert, Model 100-800). The acid-treated CC (ACC) was prepared by immersing the clean CC in 6 M HNO_3_ at 80 °C for 3 h, followed by washing with DI water and drying at 60 °C overnight. F-doped CC (FCC) was prepared by an electrochemical method wherein the clean CC was immersed in 20% HF solution and a voltage of 10 V for 15 min was applied at room temperature, followed by washing with DI water and drying at 60 °C overnight.^36^ For chitosan-coated carbon cloth (CCC), the first step is to prepare the 1% chitosan solution, that is, dissolving chitosan (100–300 K, Acros chemicals) in a 2% (v/v) acetic acid solution. Then, the cleaned CC (∼0.5 g) was immersed in 40 mL of 1% chitosan solutions for 1 h with continuous slow stirring. The obtained CCC was rinsed several times with DI water and stored for drying at room temperature.^37^ N-doped carbon cloth (NCC) was prepared as follows: first, 50 mg of dopamine hydrochloride was dissolved in 100 mL of 10 mM Tris-hydrochloride solution (pH = 8.5). Then, the CC was dipped into the solution, followed by stirring for 8 h at room temperature. The samples were then washed with DI water and dried at 80 °C overnight, followed by heat treatment at 800 °C for 2 h under nitrogen.^38^ In addition, to compare the effects of different surface charges or functional groups on the surface of various substrates, graphite sheets (GS) and nickel foam (NF) were employed. The purchased GS and acid-washed (1 M HCl, to remove the oxide layer) NF electrodes were sonicated in DI water for 30 min and finally dried overnight in an oven at 60 °C in ambient conditions.

### 2.2 Preparation of Mn-Based Prussian Blue Analogue on Various Substrates

Mn-based Prussian blue analogues (MPBs) were directly deposited on the substrates in this work by using a simple thermal deposition method.^39^ In a typical process, first, a piece of a substrate (ACC, FCC, CCC, NCC, GS, and NF, respectively, ∼4 × 4 cm^2^) was immersed in 20 mL (10 mM) of K_3_Fe(CN)_6_ solution for about 3 h in a beaker. Then, 40 mL (10 mM) of manganese(II) acetate (Mn(CH_3_COO)_2_) solution was added dropwise into the former, followed by stirring for 10 min at room temperature. Then, the beaker was sealed and maintained at 60 °C for 6 h in an oven. After naturally cooling down to room temperature, the product was thoroughly washed, followed by drying in an oven at 60 °C overnight in the air. The obtained materials decorated on various substrates are designated as MPB-ACC, MPB-FCC, MPB-CCC, MPB-NCC, MPB-GS, and MPB-NF, respectively.

### 2.3 Characterizations

The morphologies and microstructure of the synthesized materials were studied using field emission scanning electron microscopy (FESEM, ZEISS, Ultra 55) working at 10 kV. X-ray diffraction (XRD) patterns were collected in a diffractometer (XRD, PANalytical X′Pert PRO, Netherlands) with a 2θ scan from 10 to 80°. Fourier transform infrared spectroscopy (FTIR, Nicolet iS10) was used to detect the functional groups on the prepared samples. Thermogravimetric analysis (TGA) was performed using TGA Q500 (TA Instruments) in synthetic air. The specific surface area of samples was estimated by N_2_ adsorption/desorption measurements (Micromeritics Gemini VII) using Brunauer–Emmett–Teller (BET) equations. Surface charge (Zeta potential) measurements were carried out using Particle sizer-DLS/Zeta potential instrument (Beckman Coulter, Delsa Nano) for film samples in an adjustable flat surface cell mode with an area of 3.6 × 1.5 cm^2^.

### 2.4 Electrochemical Measurements

The electrochemical performances of the electrode materials were studied by using a standard three-electrode configuration with 0.5 M aqueous solution of NaCl as the electrolyte, and the platinum electrode and saturated calomel electrode (SCE) as counter and reference electrodes, respectively. The total mass of the electrode (MPB + CC) was about ∼15 mg cm^–2^.

Electrochemical measurements, including cyclic voltammetry (CV) and electrochemical impedance spectroscopy (EIS), were performed using a Gamry electrochemical workstation (Interface 1010E, Gamry Instruments, USA). The specific capacitance (*C*_s_, F g^–1^) of the electrodes was calculated according to the following equation from the CV measurements
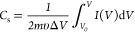
1where *m* (g) is the mass of active electrode material, ν (mV s^–1^) is the scan rate, Δ*V* (V) is the potential window, and  is the area under the CV curve. Single-frequency impedance measurement at 0.1 Hz in 0.5 M NaCl solution was used to study the surface charge based on the minimum value of the normalized capacitance calculated according to the following equation
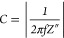
2where *f* is the applied frequency and *Z*″ is the imaginary part of the impedance.

### 2.5 Desalination of Brackish Water

Anisotropic devices were constructed with Mn-based Prussian blue analogue-modified carbon cloth as one of the electrodes (ACC being the counter electrode), where both the electrodes were directly attached to graphite current collectors to form a CDI cell.^36,40^ The desalination process was studied using a flow-through setup in single-pass (continuous) mode operations.^12^ The unit cell was fixed with plexiglass on both sides, which has a flow channel in the center to let the solution run through, by following a work described elsewhere.^41^ Electrodes were separated by filter paper to avoid electrical short. For each run, the NaCl solution (1000 ppm, 2 L) was continuously pumped into the cell at a flow rate of 5 mL min^–1^. During the measurement, the potential was kept at a constant 1.0 V for 6 min to carry out electrosorption of ions using a DC power supply (Keithley 2110), meanwhile detecting the current flow. The changes in concentration were monitored in the exit orifice of the device using an ion conductivity meter (eDAQ, EPU357, ET908).

The salt adsorption capacity (SAC, Γ, mg g^–1^) and the charge efficiency (Λ) were calculated using the following equations
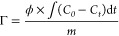
3
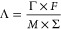
4where ϕ (L s^–1^) is the volume flow rate, *C*_0_ and *C*_*t*_ (mg L^–1^) are constant initial and final concentrations in each desalination cycle, respectively; *t* (s) is the desalination time, *m* (g) is the total mass of both electrodes, *F* is Faraday’s constant, *M* (g mol^–1^) is the molar mass for NaCl, and Σ is the charge obtained from integrating the current during the desalination process.

## 3 Results and Discussion

### 3.1 Characterizations of the MPB-Decorated Electrodes

**Figure 1:**
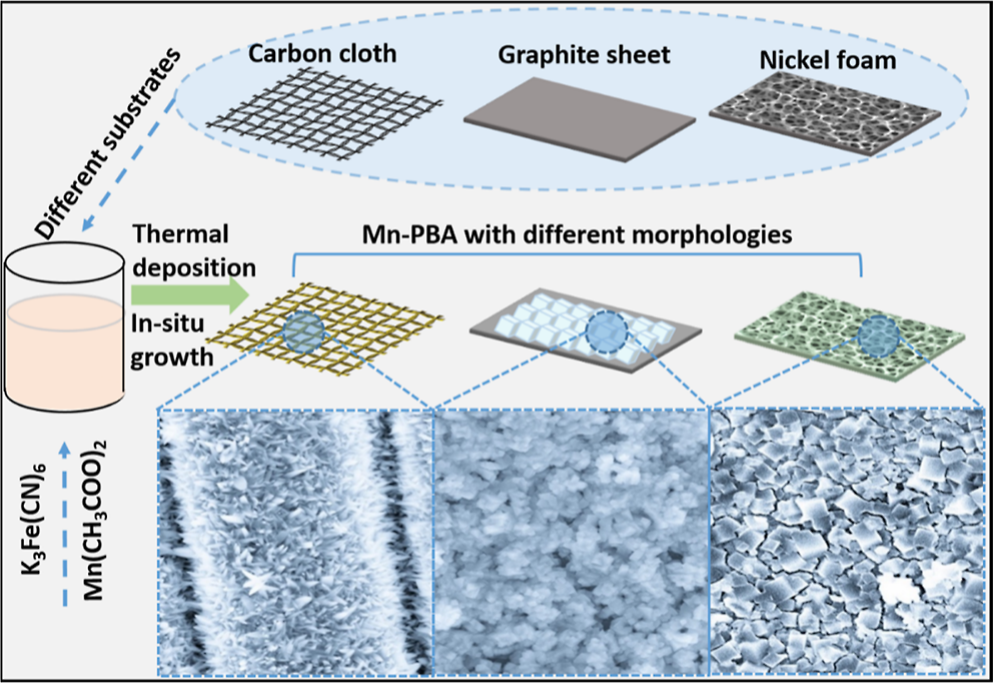
Schematic illustration of the synthesis process of MPB decorated on various substrates.

MPB directly decorated on various substrates was synthesized by using a thermal deposition method, and the approximate formation process is straightforward as schematically represented in Figure 1. First, the substrates were cleaned to remove contaminants, organic impurities, or oxide layer on the surface to improve the conductivity, enhance the surface adhesion, and increase surface-active sites, thus providing a good substrate to induce the deposition and growth of nanomaterials. The clean substrates were then immersed in Mn^2+^ solution for about 3 h for uniform Mn^2+^ adsorption on the surface. Next, the solution containing [Fe(CN)_6_]^3–^ was added dropwise to react with Mn^2+^ for in situ crystal nucleation of MPB. It is worth noting that in this process, the rate of [Fe(CN)_6_]^3–^ addition directly affects the rate of precipitation reaction, which determines the size of the final product. The faster the mixing of the two solutions, the faster the precipitation and smaller particles are formed.^39,42^ In addition, in this process, the surface of various modified substrates have different chemical bonds, functional groups, and surface charges and therefore have different binding sites and compatibilities with metal ions, resulting in slightly different morphologies and sizes of MPB nanomaterials obtained on the electrodes. Finally, the system was kept at 60 °C to provide a stable and facile thermal adsorption, thermal deposition, and crystal growth process. After naturally cooling down to room temperature, MPB nanomaterials deposited on various substrates were obtained.

**Figure 2:**
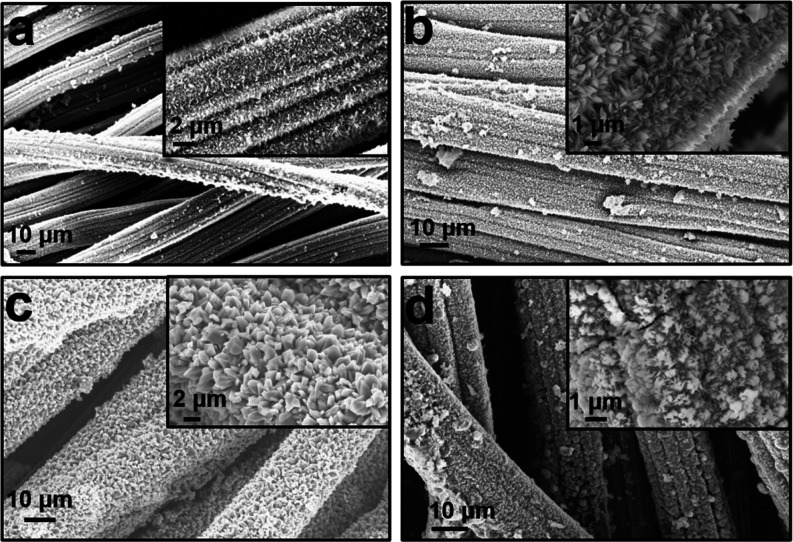
SEM images at different magnifications: (a) MPB-ACC, (b) MPB-FCC, (c) MPB-CCC, and (d) MPB-NCC, respectively.

The morphologies and structure of the synthesized MPB decorated on various substrates were characterized by SEM. As can be observed from the SEM images obtained with different magnifications (Figure 2), distributions of MPB in all the prepared materials are uniform and dense on the surface of the carbon fibers. Nanoneedle-like morphology of the MPB was found with a width of ∼200 nm and length of several micrometers in most of the surface-modified carbon substrates considered in this work (Figure 2a,b,d), except in the MPB-CCC (Figure 2c) larger particles, both in width and length being in the order of microns are observed, which may be because chitosan can provide more lone-pair electrons compared to the other substrates to “anchor” higher quantities of metal ions that could lead to secondary nucleation processes during crystal growth, generating the larger particles. It is worth noting that all the samples coated with MPB on various surface-modified carbon cloth showed similar morphology, indicating that the surface of the carbon cloth treated by different methods produced different functional groups, but they played a similar role in the process of combining with MPB. The corresponding EDX spectra (Figures S1–S4, Supporting Information) present the selected area and confirm the uniform distributions and compositions of the MPB with Mn, Fe, C, and N elements observed on differently surface-functionalized carbon cloth samples, respectively.

In contrast, MPB deposited on graphite sheet and nickel foam, with no functional groups on the surface, showed uniform nanocubic particles of relatively regular shapes, as shown in Figures S5a–c and S6a–c (Supporting Information), respectively. The cubic structured particles are dense and uniform of ∼200–300 nm diameter with an aggregated smooth surface. Notably, the particles formed on nickel foam (NF) are slightly larger, which may arise from the uncertainties in the speed of mixing the precursor solution during preparation. The corresponding EDX spectra shown in Figures S5d and S6d (Supporting Information) exhibit significant homogeneous contents of elements from the MPB with Mn, Fe, C, and N on the surface of graphite sheet and nickel foam, respectively, indicating the uniform distributions and compositions. In short, MPB with different morphologies on two different types of substrates (with/without functional groups on the surface) was successfully obtained via such a simple thermal deposition method.

**Figure 3:**
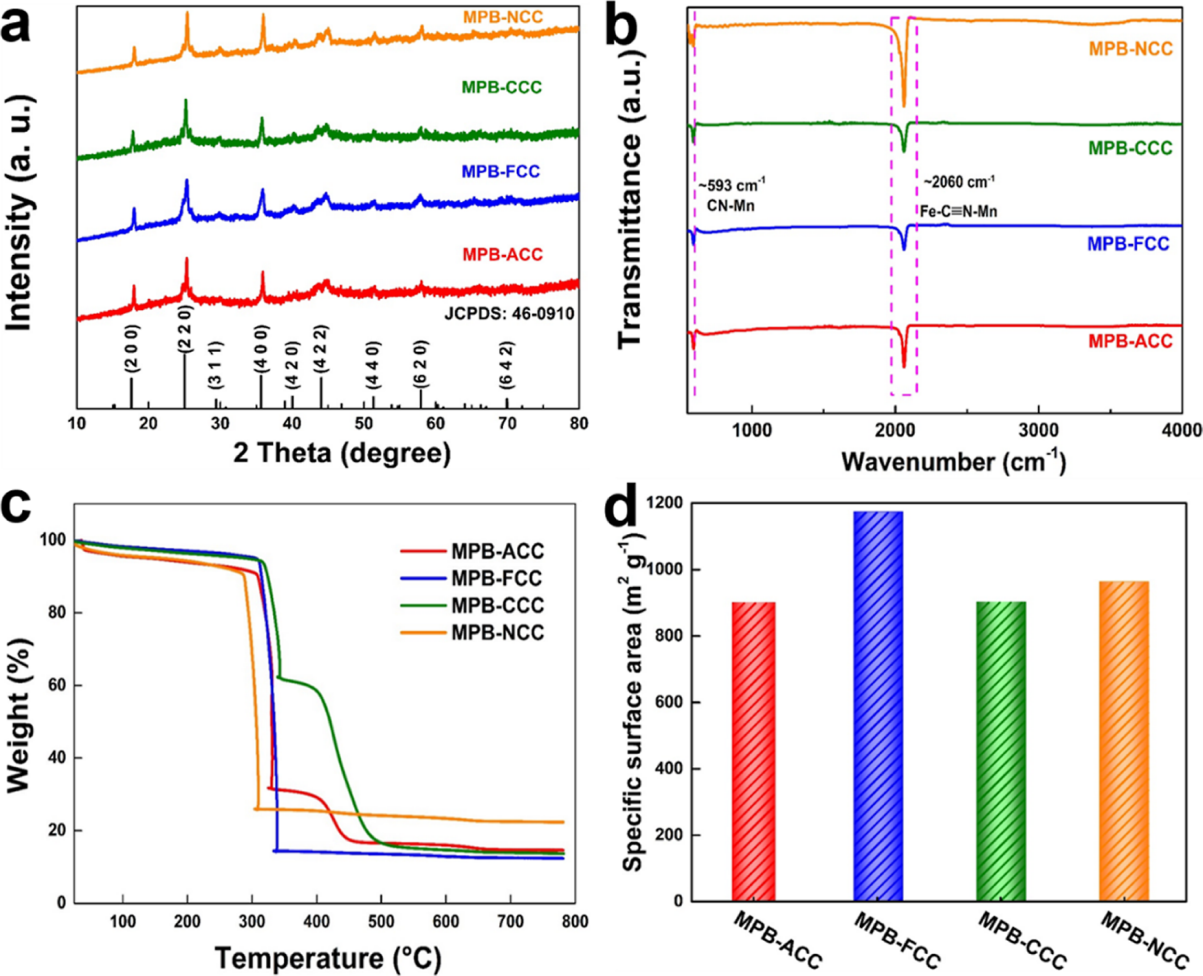
(a) XRD patterns, (b) FTIR spectra, (c) TGA characterizations, and (d) BET results of MPB decorated on various carbon cloth.

Furthermore, as shown in Figure 3a, the XRD patterns were detected to confirm the crystal phase of the materials obtained on the carbon cloth substrates. The peaks located at 2θ angles of 17.6, 25.0, 29.4, 35.7, 40.0, 44.1, 51.3, 57.9, and 69.9°, respectively, correspond to (2 0 0), (2 2 0), (3 1 1), (4 0 0), (4 2 0), (4 2 2), (4 4 0), (6 2 0), and (6 4 2) planes of Mn_2_[Fe(CN)_6_]·0.5H_2_O (JCPDS card no. 46-0910). Figure S7a (Supporting Information) shows the FTIR spectra obtained from various substrates. It is noted that the spectra of different substrates do not show any pronounced absorption peaks of compounds. These substrates processed by different materials or methods contain different functional groups, which may play different roles in the next step of loading MPB. In previous work, we have studied the surface functionalization of carbon cloth electrodes and have reported that ACC contains carboxylic groups, FCC contains C–F bonds, CCC contains C=O and C–N bonds and hydroxyl and amino groups, and NCC contains C=N and C–N bonds.^36,43^ Obviously, CCC can provide sp^2^ O sites to adsorb cyanate ions and sp^3^ N sites to adsorb metal ions, thus having the most adsorption sites to “anchor” more metal ions, leading to bigger particles as observed in the SEM images. A large number of formed crystal nuclei accumulate together, leading to secondary nucleation at a certain temperature, thereby forming larger crystal particles. In Figures 3b and S7b (Supporting Information), we present the FTIR spectra of obtained composites to illustrate the cyanide stretching frequencies. Significantly, there is a strong peak at 2060 cm^–1^ corresponding to Fe–C≡N–Mn, which confirms the high purities of the crystals.^39^ For different substrates, there is basically no shift in peak positions. Meanwhile, another peak located at ∼591 cm^–1^ indicates the CN–Mn functional group.^44^ Here, for different substrates, the peak positions are slightly shifted. The peak positions are 593.03, 592.55, 592.06, and 591.08 cm^–1^, corresponding to MPB-ACC, MPB-FCC, MPB-CCC, and MPB-NCC, respectively. This slight blue shift reveals that the electronegativity of MPB-decorated substrate materials is slightly different supporting our hypothesis. Additionally, the MPB-NCC sample shows the strongest signal demonstrating that it has the highest loading amount of nanomaterials per unit area, possibly resulting from the stronger adhesion forces between the materials and the N-doped carbon layer due to the dopamine polymerization.

Thermogravimetric analysis (TGA) was carried out in the air to investigate the thermal stability of the synthesized materials using a high resolution (HR) dynamic mode. The HR mode can quickly increase the temperature when there is no obvious reaction and barely increases the temperature when the reaction occurs until the reaction is completed.^45^ Figure S7c (Supporting Information) shows weight loss curves for all the carbon cloth substrates and the MPB powder sample. Figure 3c presents the weight loss curves for the MPB decorated on different carbon cloth substrates. Of course, the thermal stability of MPB-decorated carbon cloth is significantly reduced with the same trend of MPB powder sample. The final weight percent difference also indicates that different substrates load different contents of materials. The MPB-ACC and MPB-CCC samples show similar changes which could be because both the ACC and CCC substrates contain a certain amount of oxygen-containing functional groups, so that the decomposition of organic functional groups from the substrate and MPB occurs in the range of 310–500 °C. Meanwhile, the samples of MPB-FCC and MPB-NCC do not follow this trend, which also means that there are no oxygen-containing functional groups, but only C–F and C=N, C–N bonds that are directly formed on the surface of the carbon fiber, respectively. Obviously, the MPB-NCC samples begin to lose weight sharply before 290 °C with the worst thermal stability amongst the samples prepared in this work, which may result from the biggest amount of MPB loading with poor thermal stability in the air, which is consistent with the results obtained in FTIR studies. The TGA analysis with HR mode reveals the thermal stability of different substrates with different chemical bonds and functional groups and the effects generated from the nanostructured materials decorating the surfaces of carbon cloth.

In addition, nitrogen adsorption measurements were carried out to study the specific surface area (SSA) of the prepared materials, as shown in Figure S7d (Supporting Information) and Figure 3d, revealing the surface-active sites of all the carbon cloth before and after modification with MPB. The SSA values are 993.94, 1059.10, 1406.24, and 1014.82 m^2^ g^–1^ for ACC, FCC, CCC, and NCC, respectively. After modification, the SSA values are 901.75, 1174.67, 902.57, and 964.33 m^2^ g^–1^ for MPB-ACC, MPB-FCC, MPB-CCC, and MPB-NCC, respectively. It can be noticed that the SSA is slightly reduced after modification with MPB except for MPB-FCC, which may be due to different particle sizes. Obviously, the MPB-CCC has the biggest particle size blocking some extra pores of CCC, leading to the prominent reduction of surface area. Thus, using the thermal deposition synthesis method, MPB could be directly decorated on various substrates for possible applications as binder-free electrodes.

### 3.2 Electrochemical Performances of the Electrodes

**Figure 4:**
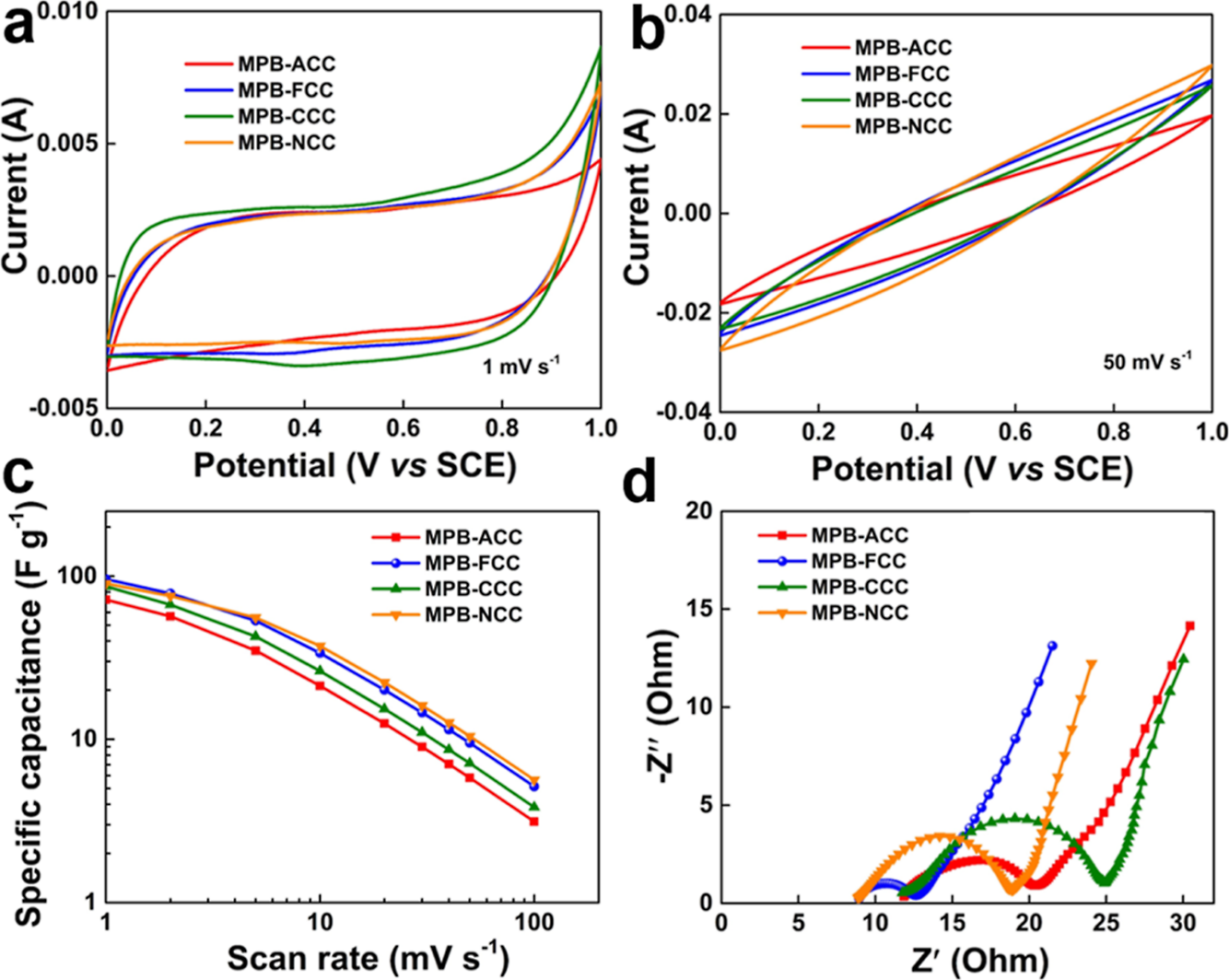
Electrochemical behaviors in 0.5 M NaCl aqueous electrolyte: (a,b) CV curves at scan rates of 1 and 50 mV s^–1^, respectively, (c) specific capacitances as a function of scan rates, and (d) Nyquist plots.

The electrochemical performances were measured to evaluate the advantages of the obtained MPB-decorated electrodes for practical application as capacitive electrodes. Figure 4a,b exhibits typical CV curves obtained at different scan rates in the potential range of 0–1 V versus SCE, respectively. As a composite electrode, the physical adsorption on the surface of the carbon fiber and the Faradaic reaction on the surface and inside the MPB material should occur simultaneously. We can observe that all curves have a pair of wide and weak peaks around 0.4 V, which is attributed to the synergistic effect. To confirm the redox reaction of the MPB material itself, we tested the electrochemical performances of MPB-GS (the capacitance generated from GS surface adsorption can be ignored). At a low scan rate of 0.4 mV s^–1^ (Figure S8a, Supporting Information), there is a pair of weak peaks appearing at ∼0.40/0.29 V and another pair of very faint peaks appearing around 0.6 V corresponding to the redox reactions of Fe and Mn elements between different valence states accompanied by the intercalation/deintercalation of Na^+^ ion. To study the reaction mechanism of the electrode, the *b*-value is evaluated from the CV results. Figure S8b (Supporting Information) shows the relationship between the log peak current and the log scan rate, corresponding to the CV curves at a fairly low scan rate from 0.4 to 2 mV s^–1^. For comparison, Figure S8c,d (Supporting Information) presents the CV test curves and the calculated b values of the same electrode in another neutral electrolyte 1 M Na_2_SO_4_. At the scan rate of 1 mV s^–1^, there are two relatively strong pairs of peaks appearing at ∼0.43/0.23 and ∼0.78/0.62 V. The slope (*b*-value) of the fitted line illustrates the mechanism of the redox reactions. When the *b*-value is close to 0.5, the redox reaction is controlled by diffusion, and when the *b*-value is close to 1, the reaction is controlled by the rapid capacitive behavior.^46,47^ Notably, the calculated result reveals capacitive kinetics of the MPB materials, indicating that the nanostructured MPB is capacitive electrode material. Figure 4b shows the CV curves of all the electrodes at 50 mV s^–1^ without obvious distortion of the redox peaks, which further indicates the capacitive behavior of the material.

As shown in Figure 4c, the specific capacitance of the MPB-FCC electrode was slightly higher (∼95.9 F g^–1^ at 1 mV s^–1^), compared to 71.7 F g^–1^ for MPB-ACC, 86.5 F g^–1^ for MPB-CCC, and 90.0 F g^–1^ for MPB-NCC electrodes. The specific capacitances of the electrodes reduce at higher scan rates, mainly due to the lower conductivity of the electrode materials and possible limited diffusion of the electrolyte ions. In addition, we can notice that the comparison trend of specific capacitance at low scanning speed is similar to the BET comparison trend of materials, which further confirms that the composite electrode exhibits obvious capacitive behavior, and the contribution of surface adsorption to the total capacitance is dominant. Figure 4d presents the Nyquist plots of all the electrodes from EIS measurements showing the conductivity and ion transport kinetics.^48^ As per these impedance plots, the *R*_s_ (the intercept at the *X*-axis at high frequency) of MPB-NCC is 8.873 Ω, which is lower than that of MPB-ACC (11.85 Ω), MPB-FCC (8.935 Ω), and MPB-CCC (11.76 Ω) electrodes, thus having the lowest intrinsic internal series resistance. The *R*_ct_ (the approximate semicircular at high to middle frequencies) of MPB-ACC, MPB-FCC, MPB-CCC, and MPB-NCC are 9.3, 3.9, 13.1, and 10.1 Ω, respectively, further confirming that the MPB-FCC electrode has the lowest charge transfer resistance and the fastest Faradic reaction rate at the interface between the electrolyte and electrode. Additionally, the *C*_L_ (the straight line at low frequency) values of all the electrodes are small, and all lines are observed significantly almost perpendicular to the *X*-axis, suggesting that the electrochemical behaviors are not controlled by diffusion but due to typical capacitive characteristics. The MPB-FCC electrode has the highest conductivity and best ion transportability, which may be due to the presence of the F-doped carbon cloth leading to a better electrochemical activity.

### 3.3 Capacitive Deionization Performances of the Electrodes

**Figure 5:**
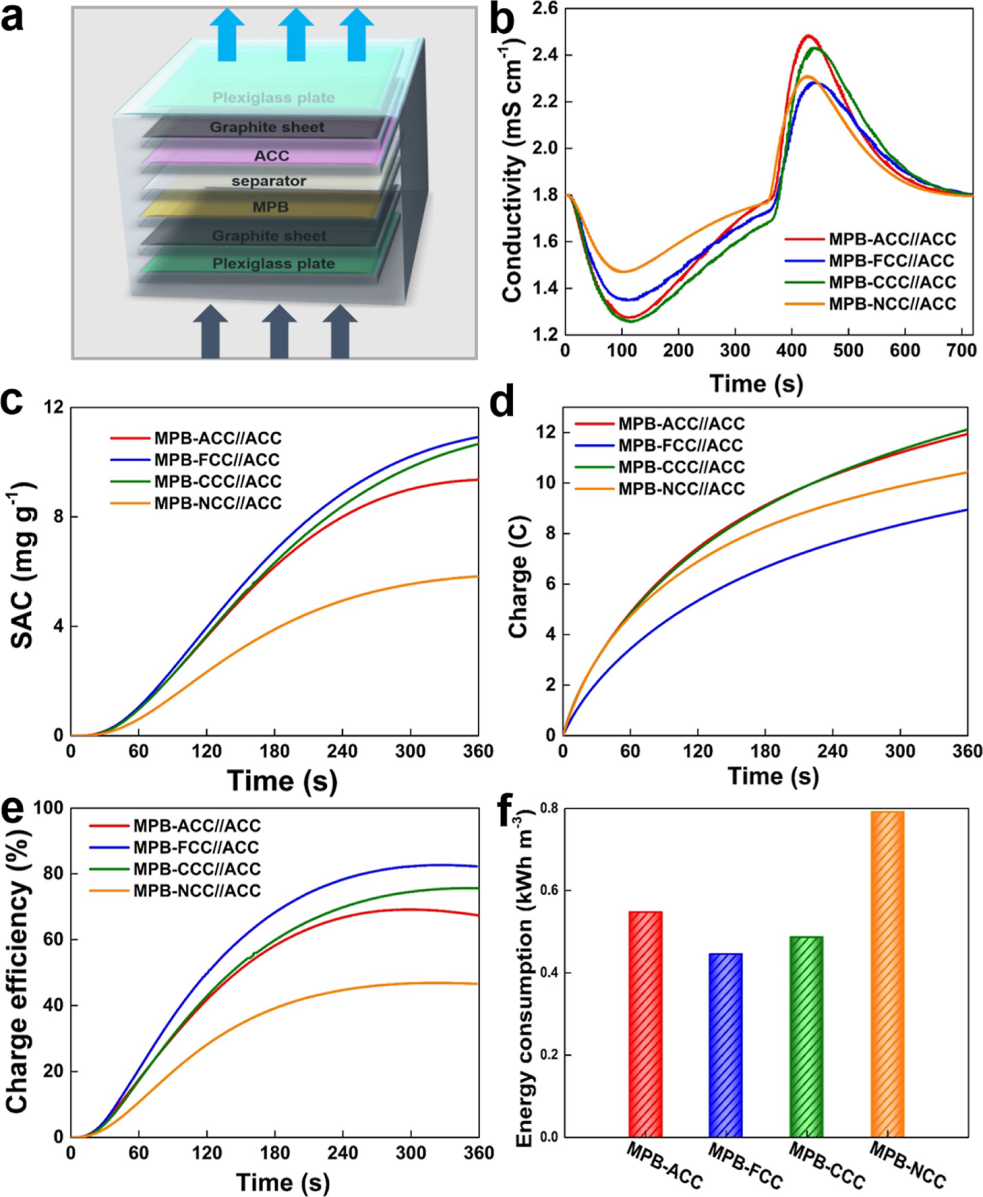
(a) Schematic diagram of device structure and the flow direction, (b) conductivity profiles, (c) salt adsorption capacitance, (d) charge curves, (e) efficiency curves, and (f) energy consumptions of different devices based on the ACC as the anode, respectively.

To study the practical applications of different MPB-decorated carbon cloth electrodes in capacitive deionization, we tested the desalination process by using an asymmetric electrode flow-through device configuration at a constant voltage (1.0 V). In all desalination processes, the electrode in the water inlet direction was the different carbon cloth electrodes decorated by MPB used as the cathode, and the electrode in the water outlet direction was ACC used as the anode (as shown in Figure 5a). Figure 5b presents the tested conductivity profiles of different asymmetric devices. Obviously, all the electrodes present a fast adsorption rate except the MPB-NCC. Additionally, in the initial stages of desalination, different electrodes show different adsorption responses as the electrochemical activities differ due to the functional groups and surface charges. In the process of intercalation desalination, compared to the slopes of the curves, it also can be noted that all electrodes almost have the same Na^+^ ion intercalation reaction rate except MPB-NCC.

The salt adsorption capacity (SAC) is an important indicator for evaluating the performance of capacitor electrodes. As shown in Figure 5c, the SAC of MPB-FCC is 10.92 mg g^–1^, whereas those of MPB-CCC, MPB-ACC, and MPB-NCC are 10.67, 9.36, and 5.83 mg g^–1^, respectively. Figure 5d shows the charging of the capacitors during ion adsorption. The charge of MPB-FCC is 8.95 C, while those of MPB-CCC, MPB-ACC, and MPB-NCC are 12.16, 11.99, and 10.47 C, respectively. Clearly, the MPB-FCC electrode has the lowest charge, which indicates a more uniform electric field distribution leading to lower energy consumption. Meanwhile, Figure 5e shows the charge efficiency of the desalination process, which can be used as an index to evaluate the effective availability of current. Same as the trend of the SAC, MPB-FCC has the highest charge efficiency around 82.28%, whereas the charge efficiencies with MPB-CCC, MPB-ACC, and MPB-NCC electrodes are 75.33, 67.05, and 46.34%, respectively. Moreover, the cycling stabilities of all these devices were tested to confirm the long-term stability for the potential of practical application (Figure S9, Supporting Information).

The energy consumption was calculated to estimate the practical applications of these electrodes as shown in Figure 5f. At the end of one desalination cycle, the energy consumption of the device (MPB-FCC//ACC) is 0.45 kW h m^–3^, whereas with MPB-CCC//ACC, MPB-ACC//ACC, and MPB-NCC//ACC, the energy consumptions are 0.55, 0.49, and 0.79 kW h m^–3^, respectively. In fact, when no MPB is deposited, the ACC symmetric device in these experimental conditions shows a SAC of 7.19 mg g^–1^, a charge efficiency of ∼54%, and an energy consumption of 0.78 kW h m^–3^. Obviously, the MPB layer provides extra desalination capacity during the Na^+^ ion intercalation process. Amongst them, MPB-FCC//ACC shows the highest salt adsorption capacity, highest charge efficiency, and lowest energy consumption. On the contrary, MPB-NCC//ACC shows the worst properties. This could be caused by the repulsive effect on cations from the positively charged NCC to reduce the total amount of Na^+^ ions adsorbed by NCC and can be inserted into MPB, resulting in a reduction in desalination capacity, a reduction in charge efficiency, and an increment in energy consumption.

According to the mechanism of Na^+^ ion intercalation, MPB-decorated carbon cloth electrodes should be used as the cathode, which can be confirmed by the results from Figure 5. From the abovementioned observation, MPB-decorated carbon cloth electrodes show completely different results when used as a cathode or anode (Figure S10, Supporting Information). It can be speculated that the most likely reason is the difference in the surface charge of the electrode. Therefore, the surface charge based on the minimum value of the normalized capacitance calculated from single-frequency impedance measurements was studied. The electrode potential of zero charge (*E*_PZC_) for each substrate is shown in Figure S11a (Supporting Information). Generally, the carboxyl group on the ACC after nitric acid treatment provides excess electrons. After fluorination, the FCC presents a large number of defects and provides numerous lone pairs of electrons due to the insertion of F atoms into the graphitic structure. After coating with chitosan, the CCC has a large number of lone pairs of electrons due to the presence of hydroxyl and amino groups but the defects are passivated by the adsorption of the polymer. In N-doped carbon (NCC), higher nitrogen content and oxygen defects occur caused by the high-temperature treatment, thus generating excess electrons resulting from the introduction of N atoms in the graphite structure. As expected, NCC shows an *E*_PZC_ of −42 mV versus SCE, which represents a significant shift to negative values compared to the untreated activated carbon cloth, indicating effective doping of the surface with positive charges, as previously reported in the literature.^49^ All other treated carbon cloth shows a significant shift to positive values compared to untreated activated carbon cloth, demonstrating effective doping of the surface with negative charges.

**Figure 6:**
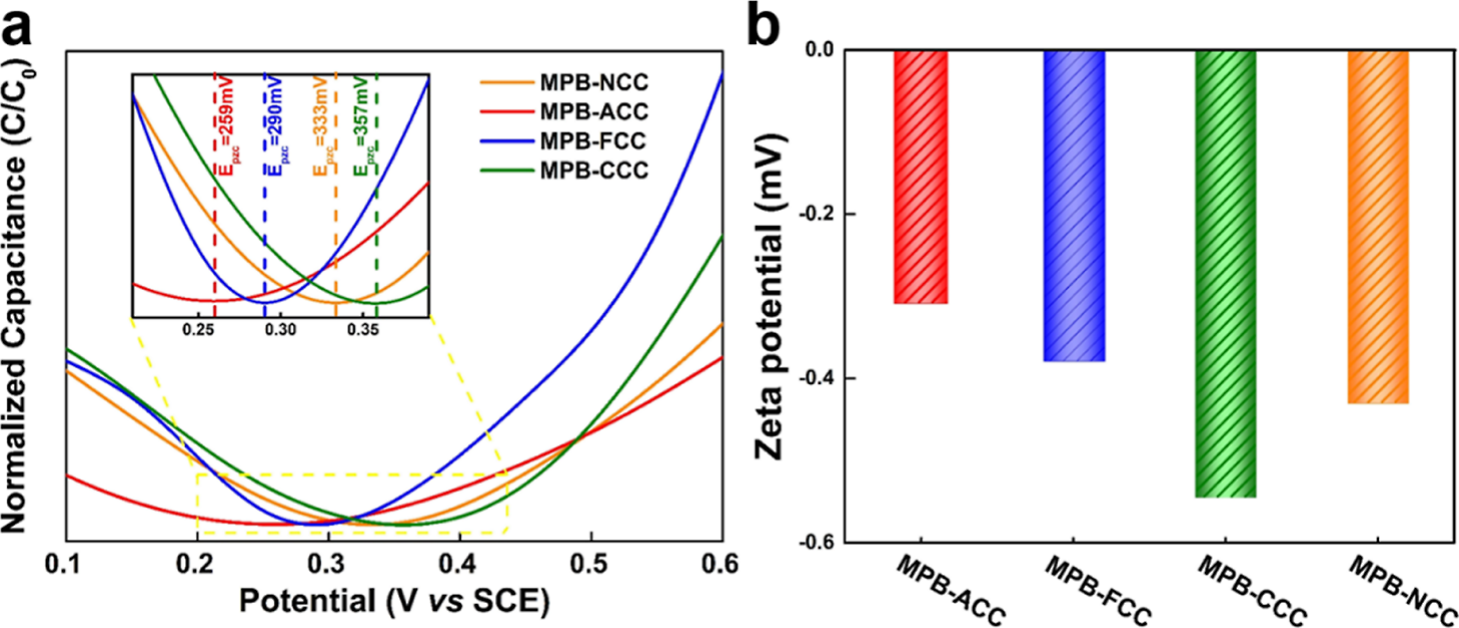
(a) Normalized capacitance obtained by impedance measurements in 0.5 M NaCl at 0.1 Hz, indicating the potential of zero charge, and (b) Zeta potential for MPB decorated various carbon cloth substrate electrodes.

The E_PZC_ of all the electrodes after decorating with MPB is presented in Figure 6a. The surface charges remain almost unchanged in the case of MPB-FCC and MPB-CCC, while MPB-NCC exhibits a large shift (291 mV) towards positive potential. It has been reported that the surface state of Prussian Blue particles in neutral media is highly negative.^50^ Thus, the large modification in surface charge between NCC and MPB-NCC indicates an enhancement in the interaction between the carbon fibers and the deposited layer. It suggests the importance of electrostatic interaction between the substrate and MPB layer in the charge transport processes. This means that the substrate largely determines the surface condition of the electrode due to the large mass ratio of the substrate electrode compared to the MPB coating, so that the composite electrode still shows similar properties to the substrate, while the MPB only weakly decreases the difference between the substrate electrodes.

Furthermore, the Zeta potential was directly tested to illustrate the surface charge of all the electrodes by using the flat surface cell. Although the test results may be inaccurate due to the excellent adsorption characteristics of carbon cloth, we can still make a slight comparison. As shown in Figure S11b (Supporting Information), we can observe that all the substrates have a negative surface charge except the NCC, which is consistent with the result shown in Figure S10a (Supporting Information). Meanwhile, the FCC has the greatest negative surface charge. After MPB modification, as shown in Figure 6b, MPB-CCC has the largest negative surface charge. Meanwhile, we can notice that after loading the negatively charged MPB on the surface, MPB-NCC shows a negative surface charge compared to the positively charged NCC. All the other electrodes have similar trends before and after MPB modification. This further proves that the surface charge caused by the electronegativity difference of each electrode is different, and the surface charge has a certain influence on the preparation and performance of the materials, which is consistent with the results described previously in this work.

Moreover, we explored the electronegativity leading to this result. The absolute electronegativities of these elements are 6.27 (C), 7.3 (N), 7.54 (O), 10.41 (F), 7.18 (H), 3.72 (Mn), and 4.06 (Fe) eV, respectively.^51^ Generally, when heteroatoms are introduced, carbon surfaces will show polarity due to the difference in electronegativity. When the electronegativity of the introduced heteroatom is higher than that of carbon, a negative surface charge is generated on a carbon surface.^52,53^ These negative surface charges on electrodes can improve cation adsorption (such as Na^+^) and desorption of anions.^36,53^ According to the different functional groups and electronegativity of each substrate, as well as our actual experimental results, we can estimate that the difference in the preparation and application of each electrode is largely affected by the surface charge. Furthermore, in our previous work, the FCC electrode presented an increase in charge carrier density due to the insertion of F atoms into the graphitic structure, inducing a larger amount of defects that provide a higher number of lone-pair electrons to promote the adsorption of Na^+^, thus boosting the salt adsorption capacity.^36^ Obviously, in this work, the MPB-FCC electrode with C–F bonds by fluorination has the highest electrochemical activity and strongest surface adsorption due to the synergistic effect of the electronegativity of multiple elements and different chemical bonds, which leads to the formation of dominant negatively charged surface and different electric field distributions on the electrode, providing improved electrochemical behaviors and enhanced CDI performances. Additionally, it also indicates that the carbon cloth surfaces treated by different methods have different functional groups and electrochemical activities, resulting in different promotion and catalytic effects when combined with the second-phase intercalation electrode material. Even though the material has opposite electronegativity to carbon cloth, the composite can bind more strongly, which would be beneficial for practical electrochemical applications.

## 4 Conclusions

A universal, facile, and simple thermal deposition strategy was investigated to obtain a novel and hierarchical Mn-based Prussian blue analogue directly decorated on various substrates. Combined with the advantages of both the surface functionalization and the open-framework materials, the composite has good electrochemical activity and physicochemical stability, high specific capacitance, and fast ion adsorption/desorption responses, thereby improving water desalination capacity. Asymmetric devices were fabricated to study the practical application. When ACC was used as the anode, the device of MPB-FCC//ACC has the highest salt adsorption capacitance (10.92 mg g^–1^), the highest charge efficiency (82.28%), and the lowest energy consumption (0.45 kW h m^–3^). Meanwhile, this work explores the effects of surface charge on capacitive material properties in capacitive deionization. It is expected that this work will provide new insights into further exploration of the relationship between second-phase materials and carbon cloth, which would offer certain guidance on the design and preparation of materials for CDI and beyond.

## Supporting Information Available

Complete details about the characterization of the materials in this work, including SEM images with EDX mapping, FTIR, TGA, and BET values; additional electrochemical performances; conduction profiles, comparison of energy consumption, and lifetime analysis of different asymmetric devices fabricated with various electrode materials for capacitive deionization; and additional impedance measurements and Zeta potential of different carbon cloth substrates (PDF)
