# Supplementary material for: Effect of Surface Charge on the Fabrication of Hierarchical Mn-Based Prussian Blue Analogue for Capacitive Desalination
Source: ACS Appl Mater Interfaces. 2022 Aug 25;14(35):40371–81. doi: 10.1021/acsami.2c08192 (PMC9460436; doi:10.1021/acsami.2c08192)
Supplement: Supplementary file 1 — am2c08192_si_001.pdf [file am2c08192_si_001.pdf]

Supporting Information

Effect of Surface Charge on the Fabrication of Hierarchical Mn-Based Prussian Blue Analogue for Capacitive Desalination

Xingyan Zhang, Esteban Toledo Carrillo, Dongkun Yu and Joydeep Dutta*

Functional Materials, Department of Applied Physics, School of Engineering Sciences, KTH Royal Institute of Technology, Hannes Alfvéns väg 12, 114 19, Stockholm, Sweden

* E-mail of corresponding author: joydeep@kth.se

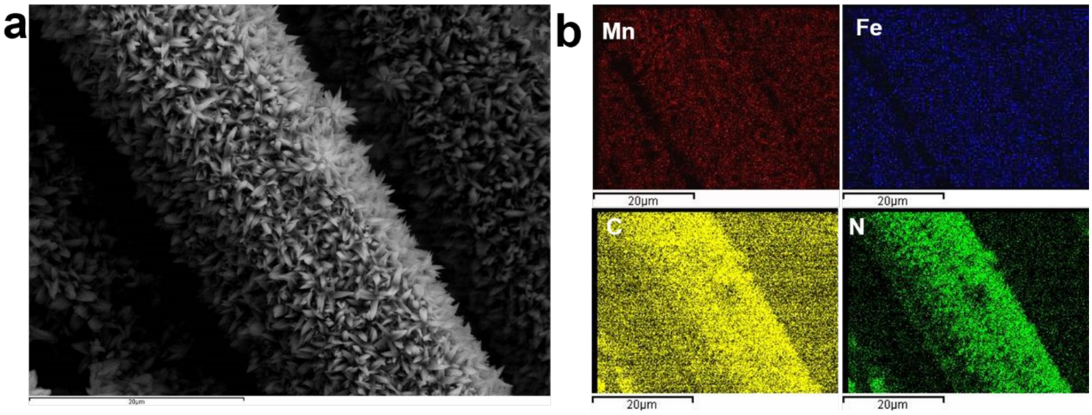

Fig. S1 (a) SEM image of the MPB-ACC, and (b) the corresponding EDX mapping.

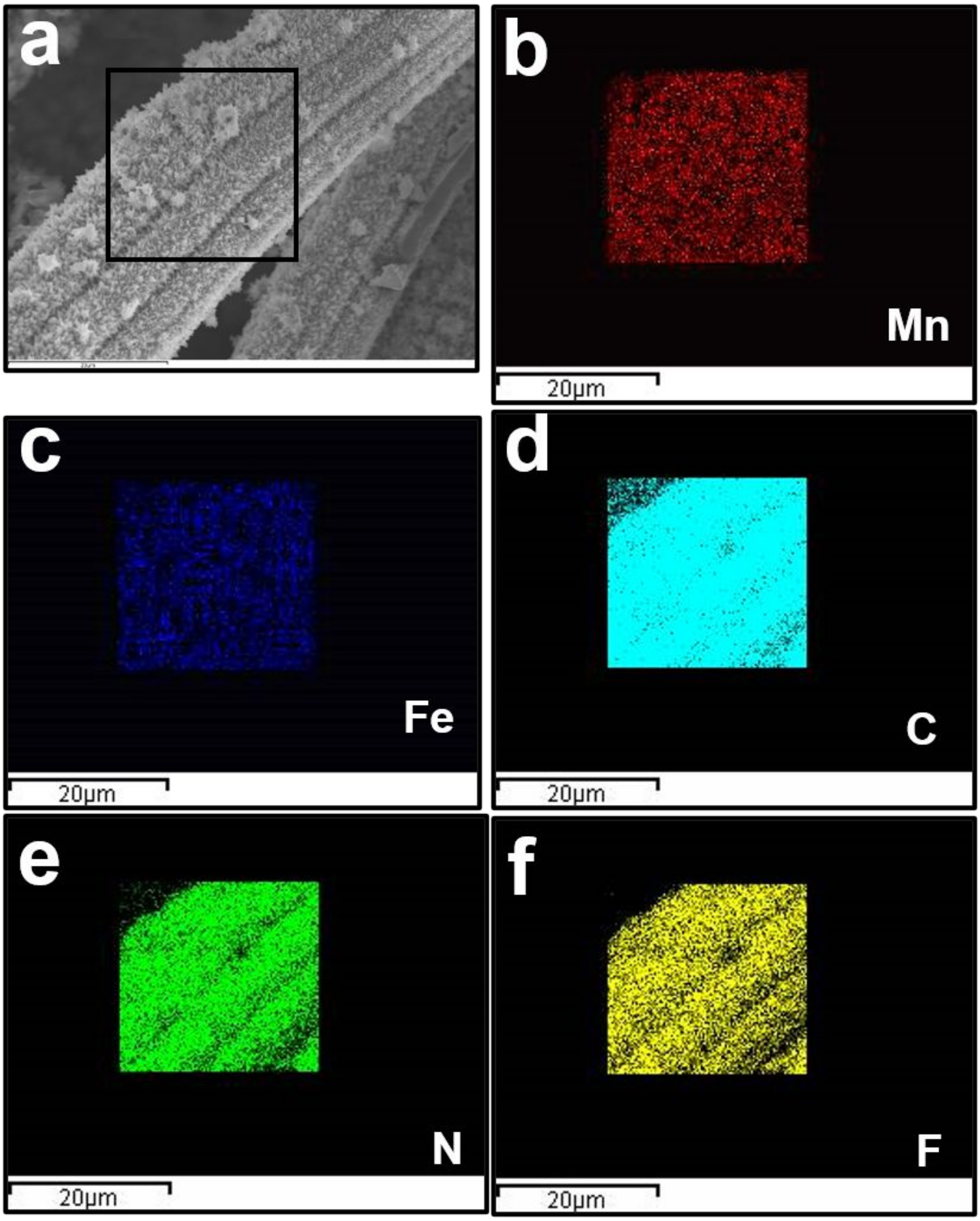

Fig. S2 (a) SEM image of the MPB-FCC, and (b-f) the corresponding EDX mapping.

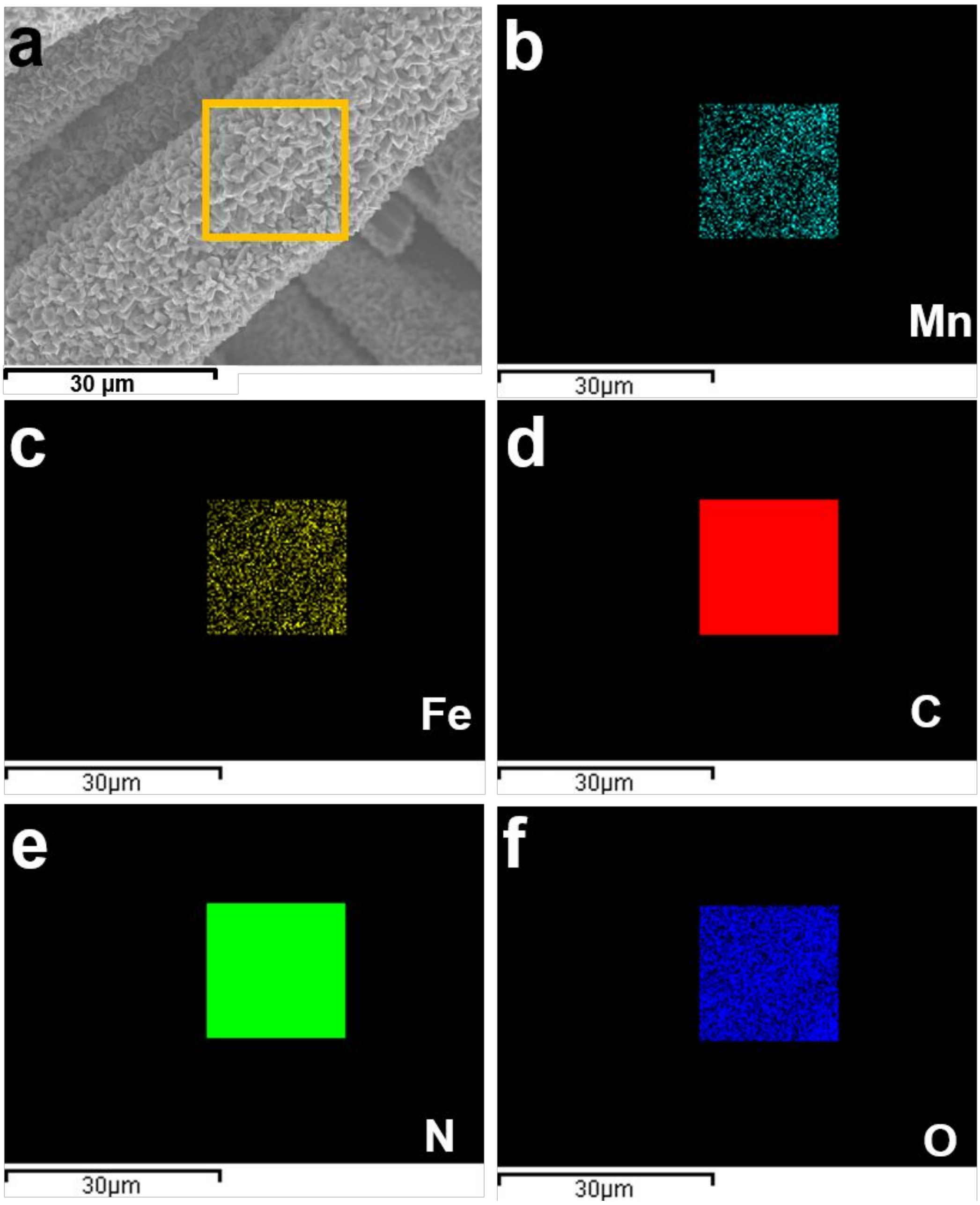

Fig. S3 (a) SEM image of MPB-CCC, and (b-f) the corresponding EDX mapping.

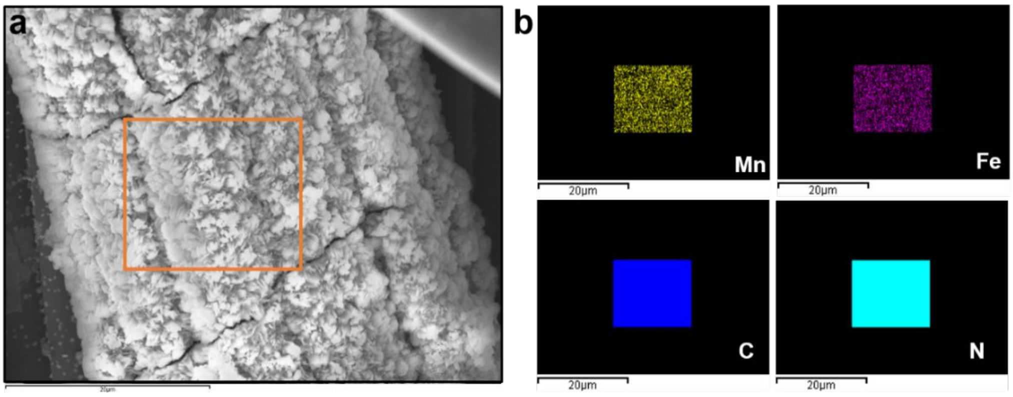

Fig. S4 (a) SEM image of the MPB-NCC, and (b) the corresponding EDX mapping.

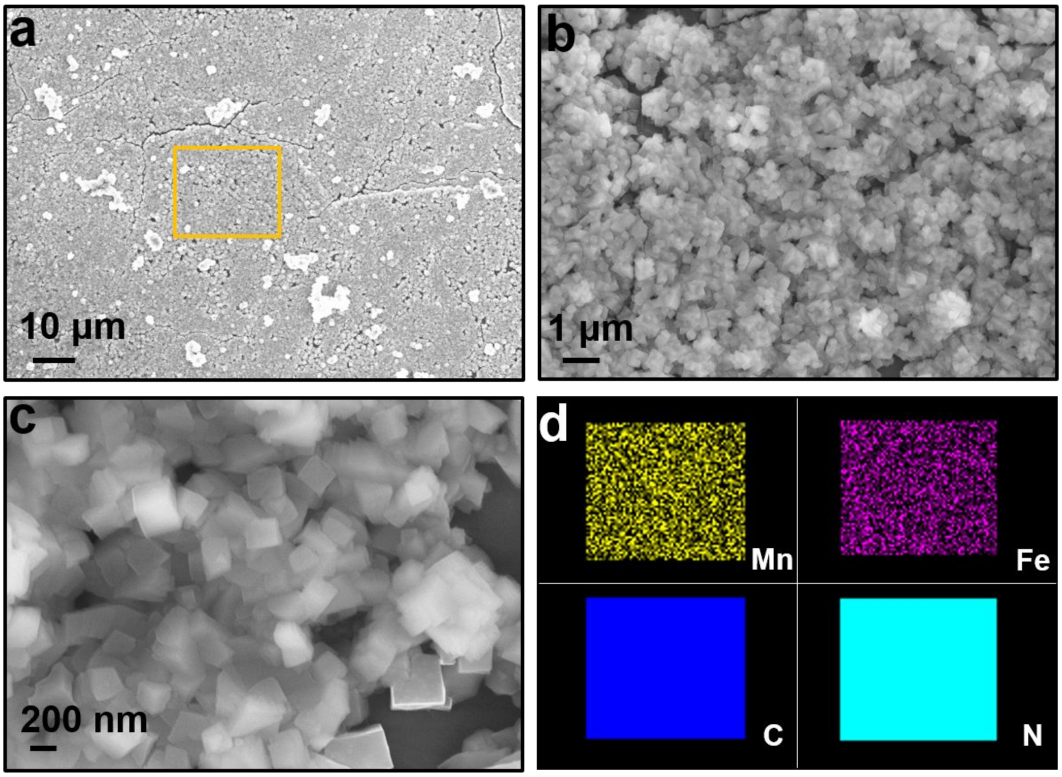

Fig. S5 (a-c) SEM images at different magnifications of MPB-GS, and (d) the corresponding EDX mapping.

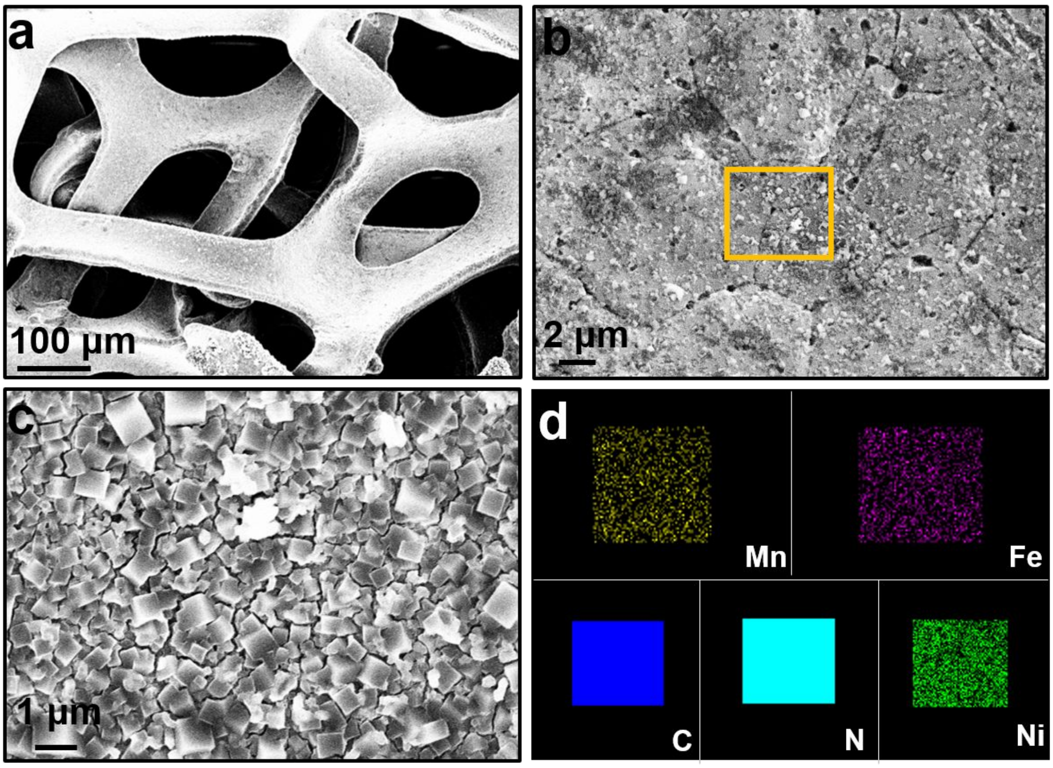

Fig. S6 (a-c) SEM images at different magnifications of MPB-NF, and (d) the corresponding EDX mapping.

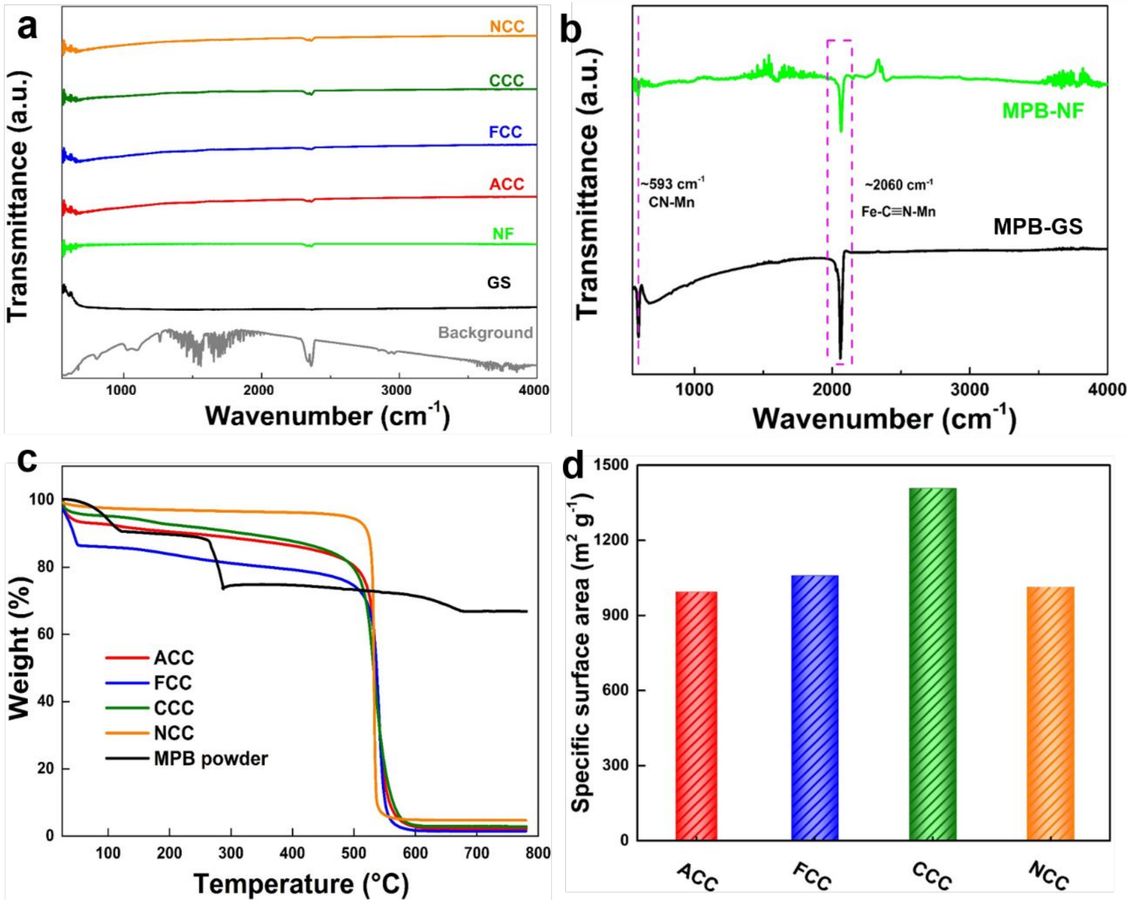

Fig. S7 (a) FTIR spectra of various substrates, (b) FTIR spectra of MPB-GS and MPB-NF, (c) TGA files of various substrates and Mn-Fe Prussian bule analogue powder, and (d) BET values of various substrates.

The TGA measurement was carried out in the ambient to investigate the thermal stability of synthesized materials by using a high resolution (HR) dynamic mode. Fig. S7c shows weight loss curves for all the functionalized carbon cloth substrates and the MPB powder sample. The weight loss curves show initially slow weight loss (< 20 wt%) below 495 °C corresponding to loss of water molecule and decomposition of the oxygen-containing functional groups on the surfaces of carbon fibers. At about 530 °C, there is a sharp weight loss of all the carbon cloth, which is due to the oxidative degradation and decomposition of carbon fiber and the removal of residual oxygen functional groups.[43] Obviously, NCC has the best thermal stability probably due to the carbonization under a high temperature. Meanwhile, the MPB powder sample shows an initially significant weight loss (~ 10 wt%) below 120 °C resulting from the water and impurities in the adsorbed air; a weight loss from 260 to 290 °C is due to the loss of coordinated water

molecules; the weight loss is caused by the oxidative degradation of carbon-containing functional groups from 290-670 °C; the complete phase transition of manganese iron oxide compounds occurs around 670 °C.

Fig. 3c presents the weight loss curves for the MPB decorated on different carbon cloth substrates. Below 280 °C, all samples are relatively stable, with only the evaporation of water molecules and the decomposition of a small amount of oxygen-containing functional groups, resulting in a small amount of weight losses (<10 wt%). The weight of the MPB-ACC sample dropped sharply from 309 °C (90 %) to 330 °C (31.6 %), decreased to 17 % at 460 °C, and further decreased to 14.9 % at 660 with a relatively slow and steady decline and then was stable upto 800 °C with retention of about 14.6 % of the initial weight. In contrast, the weight of MPB-FCC sample dropped sharply from 315 °C (90 %) to 334 °C (14.4 %), and then slowly decreased at 650 °C (12.6 %) and then kept stable upto 800 °C with retention of ~ 12.4 % of the initial weight; the MPB-CCC sample has the same trend as the MPB-ACC sample. The weight dropped sharply from 324 (90 %) to 340 °C (62.3 %), decreased to 15% at 564 °C, and decreased to 14 % at 673 °C and then kept stable upto 800 °C with retention around 13.7%; the MPB-NCC sample has the same trend with the MPB-FCC sample. The weight dropped sharply from 288 (90%) to 310 °C (26 %), and then slowly decreased to 650 °C (22.6 %) and then kept stable upto 800 °C with retention of about 22.4 % weight.

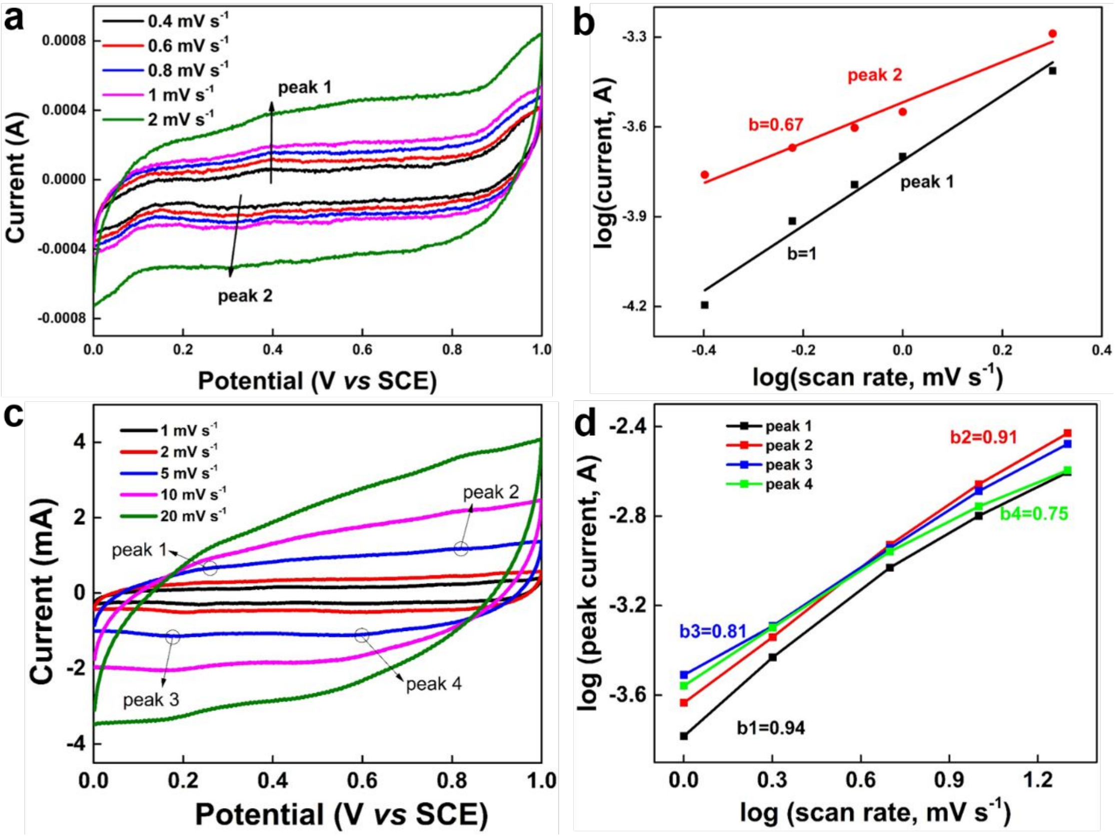

Fig. S8 (a) CV curves at different scan rates and (b) relationship between logarithm peak current and logarithm scan rate of the MPB-GS electrode in 0.5 M NaCl, (c) CV curves at different scan rates and (d) relationship between logarithm peak current and logarithm scan rate of the MPB-GS electrode in 1 M Na_2SO_4.

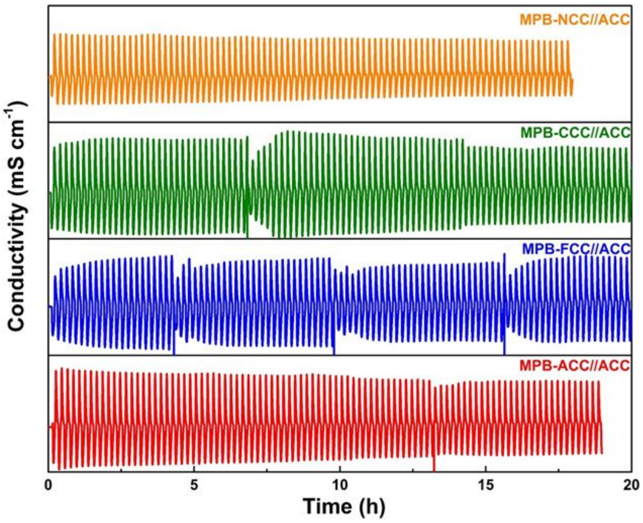

Fig. S9 The cyclic stabilities of asymmetric devices with MPB modified functional carbon cloth electrodes.

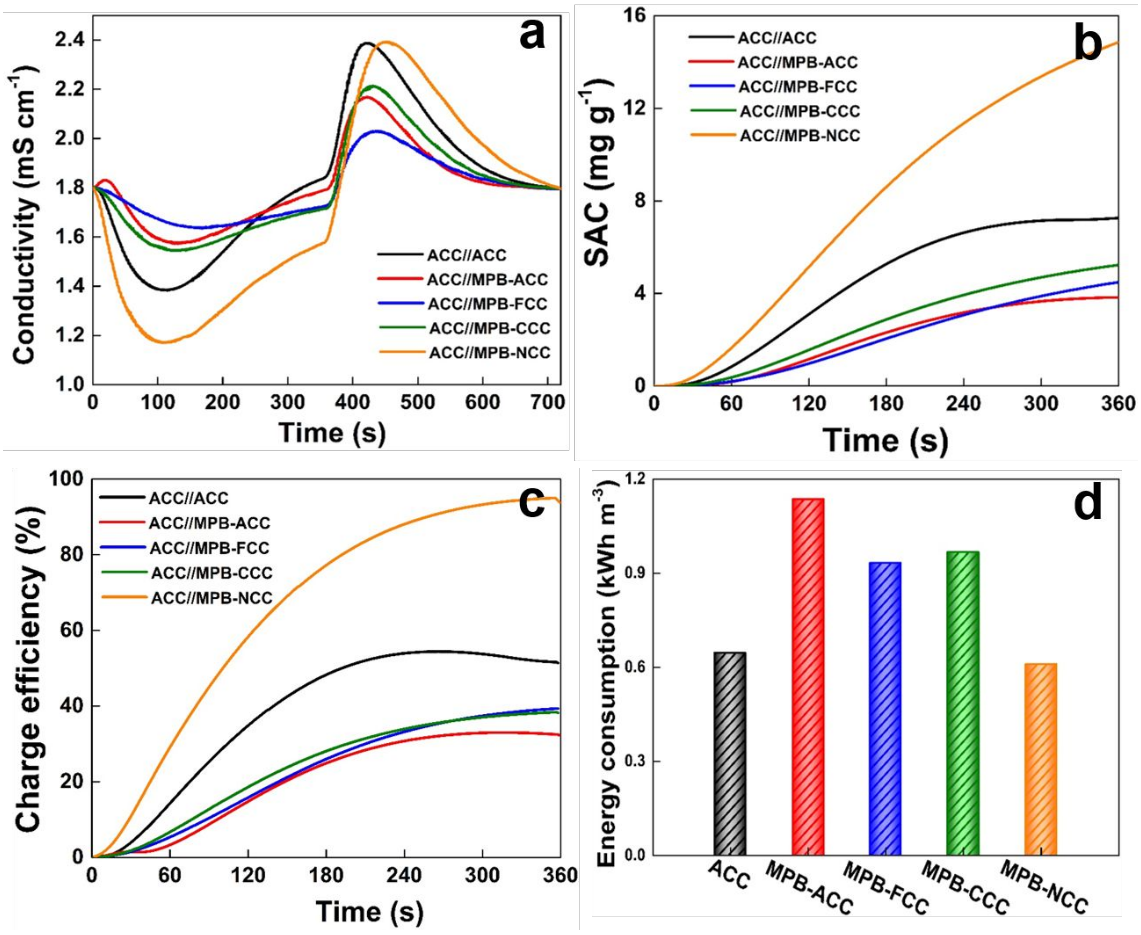

Fig. S10 (a) conductivity profiles, (b) salt adsorption capacitance, (c) charge efficiency curves, and (d) the energy consumption of different devices based on ACC as cathodes.

When the ACC was used as the cathode, in the contrary, the obtained results were different. As shown in Fig. S9a, during the process of surface adsorption and intercalation/desalination, the slope of MPB-NCC is the highest, indicating it has the fastest adsorption and reaction rate that may arise because the N-doped carbon layer coating possess positive charge, thus adsorbing more anions from the solution. As shown in Fig. S9b, the SAC of MPB-NCC is 14.86 mg g^{-1}, whereas those of MPB-CCC, MPB-FCC, MPB-ACC, and ACC are 5.23, 4.48, 3.83, and 7.26 mg g^{-1}, respectively. Obviously, although all electrodes have lower SAC than ACC except MPB-NCC. As shown in Fig. S9c, the MPB-NCC electrode has the highest charge efficiency even up to 93.69 %, while that of MPB-CCC, MPB-FCC, MPB-ACC, and ACC are 37.90, 39.31, 32.31, and 51.40 %, respectively. Furthermore, the energy consumption (shown in Fig. S9d) of the

device (ACC//MPB-NCC) is 0.36 kWh m^{-3}, whereas that with ACC//MPB-CCC, ACC//MPB-FCC, ACC//MPB-ACC and ACC//ACC are 0.97, 0.93, 1.14 and 0.65 kWh m^{-3}, respectively. Obviously, the MPB-NCC//ACC has the highest salt adsorption capacitance, highest charge efficiency and lowest energy consumption.

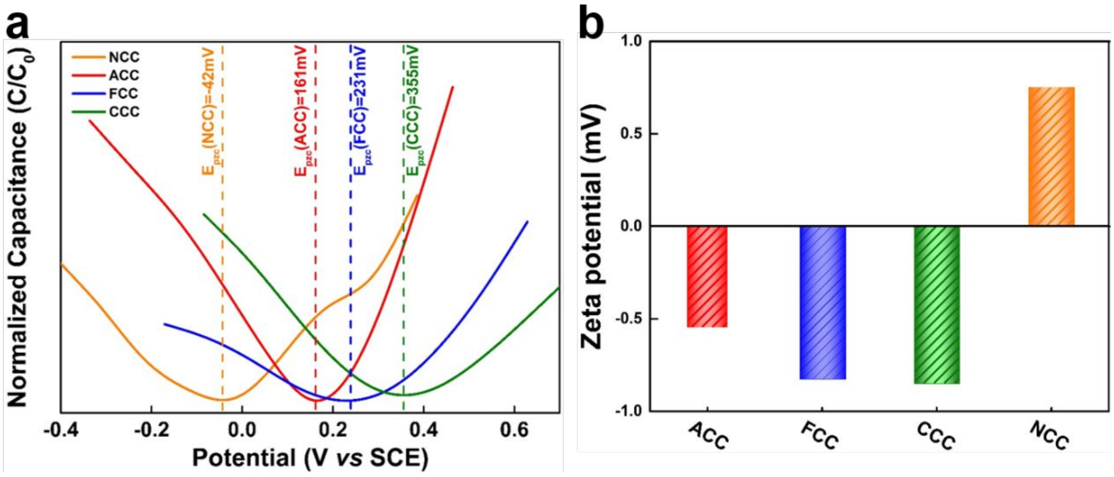

Fig. S11 (a) Normalized capacitance obtained by impedance measurements in 0.5 M NaCl at 0.1 Hz, indicating the potential of zero charge for carbon cloth substrates, and (b) Zeta potential of different carbon cloth substrates.
